# Survival of endodontically treated permanent teeth among children: a retrospective cohort study

**DOI:** 10.1186/s12903-021-01952-y

**Published:** 2021-11-19

**Authors:** Saitah Bufersen, Judith Jones, Jayapriyaa Shanmugham, Tun-Yi Hsu, Sharron Rich, Ali H. Ziyab, Sami Chogle

**Affiliations:** 1 Department of Endodontics, Boston University Henry M Goldman School of Dental Medicine, 635 Albany St, G200, Boston, MA 02118 USA; 2 University of Detroit Mercy School of Dentistry, Detroit, MI USA; 3 Department of Community Medicine and Behavioral Sciences, Faculty of Medicine, Kuwait University, Kuwait City, Kuwait

**Keywords:** Endodontically treated teeth, Root canal therapy, Survival analysis, Children

## Abstract

**Background:**

Outcome studies of nonsurgical root canal treatment (NSRCT) in permanent teeth of children are scarce. This study investigated survival and assessed the variables associated with failure of endodontically treated teeth (ETT) in 6- to 18-year-olds.

**Methods:**

Records of subjects who received NSRCT at age 6–18 years at Boston University between 2007 and 2015 were assessed for the occurrence of untoward events. Kaplan–Meier survival curves were used to investigate the survival of ETT in the total sample. Adjusted hazard ratios (aHRs) and 95% confidence intervals (CIs) were estimated.

**Results:**

The analysis included 341 patients (424 ETT). Kaplan–Meier survival curves differed according to age at treatment (log-rank *P* = 0.026), with survival being the lowest among the youngest age group. The estimated 5-year survival probability was 80% for 15- to 18-year-olds, 64.8% for 12- to 14-year-olds and 46.4% for 6- to 11-year-olds. Compared to age at treatment of 15–18 years, age at treatment of 6–11 years (aHR: 2.19, 95% CI 1.02–4.67) and 12–14 years (aHR: 2.02, 95% CI 1.15–3.55) was associated with an increased risk of ETT failure. In the total study sample, the estimated cumulative survival probability was 93.3% at 12 months, 88.0% at 24 months, 76.2% at 36 months, 71.0% at 48 months, and 69.1% at 60 months.

**Conclusions:**

In children, ETT are more likely to survive when NSRCTs are performed at an older age.

## Background

In the general population, multiple outcome studies have been performed regarding nonsurgical root canal treatment (NSRCT) with varying results. A number of factors influence this variation including the outcome measured, the study design, follow-up duration and operator skill and experience. The success rate of NSRCT ranges from 75 to 85%, depending on the strictness of the criteria used [1]. The percentage of healed teeth ranges from 73 to 97%, while functional teeth range between 88 and 97% [2].

Few studies have investigated endodontic involvement and NSRCT in the permanent teeth of children. In a Turkish population, evaluation of panoramic radiographs and charts of 6- to 12-year-olds showed that 0.47% of permanent first molars were endodontically treated, while 4% required NSRCT [3]. In 13- to 16-year-olds, 4.28% of permanent first molars were endodontically treated and 6.09% required NSRCT [3]. Another study also examined patient charts in a Saudi population and reported that 35.8% of permanent teeth in 6- to 18-year-olds were pulpally involved [4].

Reports on the NSRCT outcomes in permanent teeth of this younger population are rare [5]. The technical quality of NSRCT was satisfactory in only 42–61% of cases, with a mean age of 12–16 years at treatment time [6–8]. Radiographic exams of endodontically treated teeth (ETT) showed healthy periapical tissue in 48–75% of teeth [6, 8]. Moreover, successful NSRCT was reported in 36–86% of teeth in 8- to 20-year-olds after clinical and radiographic exams [5, 9]. The marked difference in the previous studies' results could be attributed to differences in design and the criteria used to measure the outcome. The purpose of this study was to examine the survival rates of NSRCTs performed on permanent teeth of 6- to 18-year-olds at Boston University Henry M. Goldman School of Dental Medicine (BUGSDM). Predictors of failure of ETT were also assessed. Our null hypothesis was that age, gender, insurance type, tooth type and jaw type have no effect on treatment outcome (survival).

## Methods

### Study design, setting, and participants

**Fig. 1:**
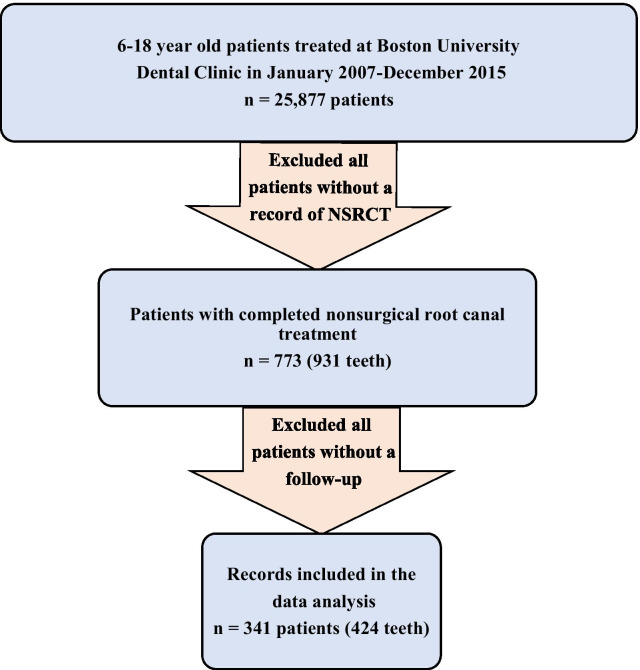
Flow chart of the records included in the study

In this retrospective analysis of electronic dental records of 6- to 18-year-olds, patients were selected after ethical approval was obtained from the Medical Center Institutional Review Board (IRB Number: H-34766). A total of 25,877 records were scanned to identify 773 patients (931 teeth) who received endodontic treatment between January 1st, 2007, and December 31st, 2015. Subjects with completed NSRCTs during the study period and at least one posttreatment record were included in the analysis. Hence, records of 432 patients (507 teeth) were excluded due to lack of follow-up, leaving a sample size of 341 patients with 424 teeth (Fig. 1). The patient data were deidentified and a study ID was used to record the findings. The NSRCT was mainly performed by endodontic residents who were trained to perform microscopic endodontic therapy using evidence-based protocols and techniques with specialist faculty supervision for uniform results.

### Study outcome ascertainment

The main outcome variable was time until failure of ETT, i.e., time-to-event or time until a participant has the event of interest. If the ETT was symptomatic or subjected to any of the following interventions: extraction, endodontic retreatment, or apical surgery, it was deemed a failure. Otherwise, survival was defined as the absence of reported clinical signs or symptoms and the absence of a treatment intervention regardless of the radiographic interpretation.

### Covariates

Data on the following covariates were extracted from the electronic dental records: age at time of treatment (6–11, 12–14, and 15–18 years), gender, insurance type (Medicaid [MassHealth] and private/self-paid), tooth type (posterior and anterior), and jaw type (maxillary and mandibular). Insurance type was used to reflect on the patient socioeconomic status (SES).

### Statistical analysis

Analyses were conducted using SAS 9.4 (SAS Institute, Cary, NC, USA). The statistical significance level was set at α = 0.05. Descriptive analyses were conducted to calculate frequencies and proportions of categorical variables. Continuous variables were described by estimating their median and related interquartile range (IQR). Time-to-event (i.e., time from treatment to failure of ETT) was defined as the time in months from the date of NSRCT completion to the date of ETT failure. Right censoring was considered for all ETT that did not fail at their last observed follow-up time. Hence, the time in months to either ETT failure or censoring was calculated for each ETT. Given the dynamic study design (i.e., participants may enroll at different times throughout the study period: from January 1st, 2007 to December 31st, 2015), participants who had their NSRCT before the end of the study period were followed for a shorter period than participants who had their NSRCT earlier in the study period. Hence, follow-up time (time at risk of failure after NSRCT) was not the same for all participants, and survival time for some participants was not known (censored) due to either a person not experiencing the study outcome (ETT failure) before the end of the study or a person was lost to follow-up. Thus, we applied survival analysis that considers information on event status (ETT failed vs. censored) and follow-up time to estimate survival function, i.e., the probability that a person survives past a certain time point. Kaplan–Meier (KM) survival curves were constructed to show the cumulative risk of ETT failure in the total sample and according to categories of the following variables: age at time of treatment, gender, insurance type, tooth type, and jaw type. The log-rank test was applied to assess whether the KM survival curves of two or more independent groups were statistically equivalent. The Cox proportional hazards regression model was applied to assess associations between multiple predictors and the time-to-event outcome. Unadjusted and adjusted hazard ratios (HRs) and their related 95% confidence intervals (CIs) were estimated. To check that the hazards proportionality assumption of the Cox proportional hazards regression model was not violated, interaction terms between the covariates/predictors and time were assessed. An interaction term *p* value < 0.05 was used to indicate if the proportionality of hazards assumption was violated.

## Results

In total, 25,877 subjects who were 6–18 years of age attended BUGSDM clinics between January 1st, 2007, and December 31st, 2015, of whom 773 patients (931 teeth) underwent NSRCT. Of those, 341 patients (424 teeth) had at least one posttreatment record and thus were included in the analysis (Fig. 1). The analyzed sample of ETT (n = 424; i.e., teeth with at least one posttreatment follow-up) and the sample of ETT that had no follow-up information (n = 507; were not analyzed in the current report) were not significantly different in all characteristics investigated (Table 1). In the analytical ETT sample, the median age at treatment was 16.0 years (IQR: 3.0 years), and the median follow-up time was 14.0 months (IQR: 28.0 months). The majority of subjects had NSRCTs at ages 15–18 years (66.5%), followed by 12–14 years (24.5%; Table 1). There were more female than male subjects (54.9% vs. 45.1%). Most subjects had MassHealth (Medicaid) insurance (62.3%). NSRCTs were performed more often on posterior than anterior teeth (72.6% vs. 27.4%). There were slightly more NSRCTs performed on teeth located in the maxillary jaw than on those located in the mandibular jaw (52.8% vs. 47.2%).

**Table 1:** Characteristics of the endodontically treated teeth (ETT) with (analytical sample; n = 424) and without (n = 507) follow-up information

Variable	Sample of ETT with no follow-up information^a^ (n = 507)	Analytical sample of ETT^b^	
		Total analytical sample of ETT (n = 424)	Failed ETT (n = 63)
	% (n)	% (n)	% (n/total)
*Gender*			
Male	48.1 (236)	45.1 (191)	13.1 (25/191)
Female	51.9 (255)	54.9 (233)	16.3 (38/233)
*Age at treatment*			
6–11 years	13.6 (69)	9.0 (38)	26.3 (10/38)
12–14 years	31.0 (157)	24.5 (104)	22.1 (23/104)
15–18 years	55.4 (281)	66.5 (282)	10.6 (30/282)
*Tooth type*			
Posterior	79.3 (402)	72.6 (308)	16.2 (50/308)
Anterior	20.7 (105)	27.4 (116)	11.2 (13/116)
*Jaw type*			
Maxillary	47.9 (243)	52.8 (224)	13.8 (31/224)
Mandibular	52.1 (264)	47.2 (200)	16.0 (32/200)
*Insurance type*			
MassHealth	72.8 (369)	62.3 (264)	15.9 (42/264)
Private/Self-pay	27.2 (138)	37.7 (160)	13.1 (21/160)

*ETT* endodontically treated teeth

^a^These 507 teeth were not analyzed due to no follow-up information (i.e., lost to follow up after treatment)

^b^These 424 teeth had at least one posttreatment follow-up and hence, were included in the current analysis

In total, 63 ETT (14.9%) failed throughout the study period and 361 teeth (85.1%) were censored. The proportion of ETT failures was not different according to gender, jaw type, tooth type or insurance type (Table 1). However, younger age at treatment was related to a higher proportion of ETT failure, with 26.3% of ETT at age 6–11 years failing during the study period and only 10.6% of ETT at age 15–18 years failing (Table 1). Untoward events are shown in Table 2, with extraction being the most common event (68.0%). Not all of the intended treatments were performed. For instance, of the 63 ETT that failed, only 33 teeth were eventually extracted from among the 43 teeth planned for extraction. Additionally, 2 more teeth were extracted from among the 14 teeth planned for retreatment. In total, 35 teeth were extracted, and the retention rate after NSRCT was 91.7%.

**Table 2:** Record of untoward events of the endodontically treated teeth (ETT)

N = 63	Untoward event		Actual treatment received
29	Planned for extraction^a^	Non-restorable = 15	Extraction = 8
		Orthodontics = 7	Extraction = 7
		Unknown = 7^b^	Extraction = 7
24	Signs and symptoms	Extraction = 14^a^	Extraction = 11
		Retreatment = 6^c^	Retreatment = 5
		Apical surgery = 2^d^	Apical surgery = 1
		Other = 2	
8	Planned for retreatment^c^	Retreatment = 7 (1 later extracted)	
		Extraction = 1	
2	Planned for apical surgery^d^	Apical surgery = 2	

^a^33 ETT were extracted from 43 that required extraction (2 more ETT were extracted from retreatment group)

^b^ETT not present on posttreatment radiographs with no record of extraction in patient chart (extracted at another clinic)

^c^12 ETT were retreated from 14 ETT that required retreatment

^d^3 ETT had periapical surgery from 4 ETT that required surgery

**Fig. 2:**
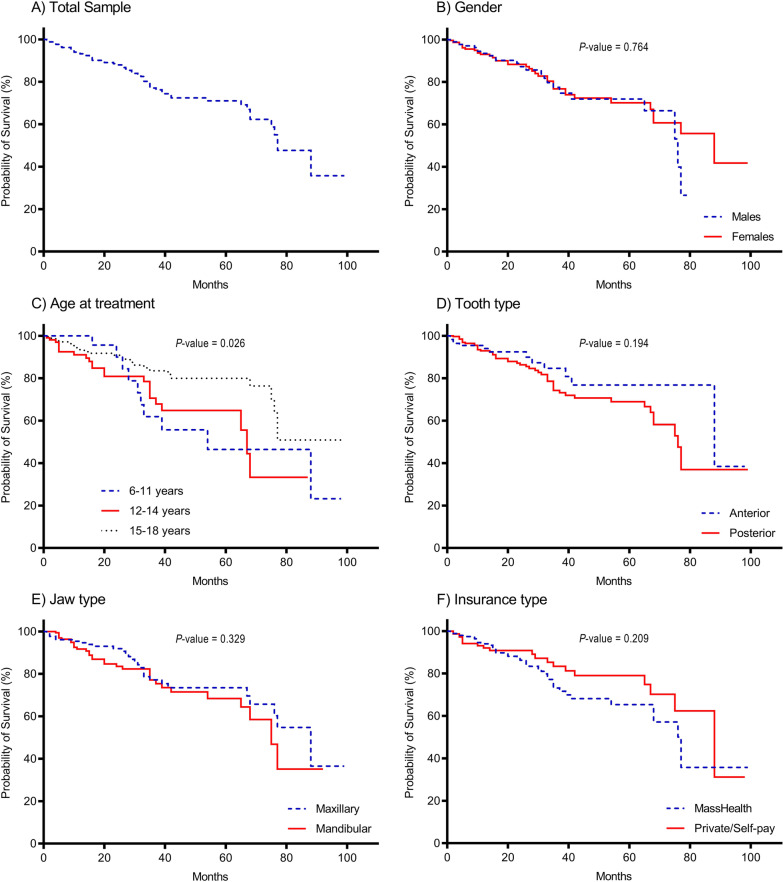
Kaplan–Meier (KM) survival curves of endodontically treated teeth (ETT) in the total study sample (**A**) and according to: **B** gender, **C** age at treatment, **D** tooth type, **E** jaw type, and **F** insurance type. Log-rank test was used to estimate the presented *p* values that compare the survival curves of independent groups

Figure 2 shows KM survival curves for the total study sample and according to the abovementioned covariates. The overall median survival time of ETT was estimated to be 77 months (6.4 years; Fig. 2A), i.e., 50% of ETT survived beyond 77 months. There was no evidence of a difference in KM survival curves of ETT according to gender (*p* value = 0.764; Fig. 2B), tooth type (*p* value = 0.194; Fig. 2D), jaw type (*p* value = 0.329; Fig. 2E), or insurance type (*p* value = 0.209; Fig. 2F). However, KM survival curves according to age at time of treatment showed differences in survival of ETT (*p* value = 0.026; Fig. 2C), with the youngest age group demonstrating the lowest rates of ETT survival. Among the 15–18 age group, the estimated cumulative survival probability after treatment was 93.2% at 12 months, 90.9% at 24 months, 83.5% at 36 months, and 80.0% at both 48 months and 60 months. For the 12–14 age group, the estimated cumulative survival probability was 91.1% at 12 months, 80.9% at 24 months, 70.6% at 36 months, and 64.8% at both 48 months and 60 months. For the 6–11 age group, the estimated cumulative survival probability after treatment was 100% at 12-months, 90.0% at 24 months, 61.9% at 36 months, 55.7% at 48 months, and 46.4% at 60 months. Among all groups, the average estimated cumulative survival probability after treatment was 93.3% at 12 months, 88.0% at 24 months, 76.2% at 36 months, 71.0% at 48 months, and 69.1% at 60 months.

Unadjusted and adjusted HRs were estimated using Cox regression models to assess associations between predictors and time to ETT failure (Table 3). There was no evidence that the proportionality of hazards assumption was violated, as indicated by statistically nonsignificant predictor-time interaction terms (*p* values > 0.05). In the unadjusted analysis, young age at treatment showed an association with an increased risk of ETT failure; this association remained statistically significant after adjustment for the effect of other predictors (Table 3). For instance, compared to age at treatment of 15–18 (reference category), age at treatment of 6–11 (adjusted HR = 2.19, 95% CI 1.02–4.67) and 12–14 (adjusted HR = 2.02, 95% CI 1.15–3.55) was associated with an increased risk of ETT failure (Table 3). None of the other assessed predictors showed associations with ETT failure. Nonetheless, posterior ETT showed a trend for a higher risk of failure than anterior ETT (adjusted HR = 1.68, 95% CI 0.81–3.45; Table 3). The difference was not statistically significant.

**Table 3:** Hazard ratios for risk of endodontic treatment failure associated with gender, age at treatment, tooth type, jaw type, and insurance type

Variables	Unadjusted model		Adjusted model	
	HR (95% CI)	*P* value	HR^a^ (95% CI)	*P* value
Gender (Male vs. Female)	1.08 (0.65–1.80)	0.765	1.09 (0.65–1.82)	0.754
Age at treatment (6–11 vs. 15–18 years)	1.87 (0.90–3.86)	0.092	2.19 (1.02–4.67)	0.043^*^
Age at treatment (12–14 vs. 15–18 years)	1.98 (1.15–3.43)	0.014	2.02 (1.15–3.55)	0.015^*^
Tooth type (posterior vs. anterior)	1.49 (0.81–2.76)	0.199	1.68 (0.81–3.45)	0.167
Jaw type (maxillary vs. mandibular)	1.28 (0.78–2.10)	0.332	0.95 (0.54–1.68)	0.869
Insurance type (MassHealth vs. private/self-pay)	1.40 (0.83–2.37)	0.214	1.17 (0.67–2.04)	0.592

*HR* hazard ratio, *CI* confidence interval

*P value < 0.05

^a^Hazard ratios adjusted for all variables shown in the table

## Discussion

This study investigated the prognosis of NSRCTs in permanent teeth of children in terms of survival, with the null hypothesis that survival is not influenced by gender, age at treatment, insurance type, tooth type, or jaw type. The results show that survival was influenced only by age at treatment, and hence we reject the null hypothesis that age at treatment does not affect the survival of NSRCTs. Among children, older age at treatment was associated with higher survival of ETT. The oldest age group had the highest 5-year survival (80.0%) compared to the youngest age group (46.4%).

In the current study, only age at treatment was significantly related to ETT survival, while gender, SES, tooth type, and jaw type showed no statistical association. A systematic review by Ng et al. concluded that there is no significant association between survival after NSRCT and gender or tooth type [10]. Dammaschke et al. and Mareschi et al. also reported no significant association between survival of ETT and gender, jaw type or tooth type [11, 12]. Kwak et al., however, reported that ETT in males was significantly more likely to end with extraction than in females [13]. They attributed this to the higher prevelence of vertical root fracture and periodontal disease in male patients. For SES, a study by Raittio et al. reported that there was no association between SES and endodontic treatment quality [14].

While the current study did not find a statistically significant association between tooth type and survival, there was a trend suggesting that anterior ETT were more likely to survive than posterior ETT. Molar ETT have been repeatedly shown to have the worst survival outcome in many studies [15–18]. This could be due to the easier access to and less complex anatomy of anterior teeth when compared to posterior teeth.

Several studies found associations between ETT survival and age. These studies have reported an increase in the failure rate with increasing patient age. Iqbal reported that the majority of failures occurred in the older age group (41–50 years) and the least in the youngest age group (21–30 years) [17]. It was suggested that the presence of calcified canals in the older age group and uncooperative behavior along with poor oral hygiene maintenance and a low literacy rate could be the causes. The aforementioned study clinically and radiographically assessed 90 patients 21–50 years of age who attended the department during a 6-month period for failed ETT. Kwak et al. have reported that ETT in younger patients were significantly more likely to survive [13]. This was attributed to the lower likelihood of vertical root fracture in younger patients and to fewer restorative interventions compared to older patients. Kwak et al. included patients of all age groups, with the youngest group being subjects less than 20 years old [13]. Lee et al. also included patients of all age groups, with the youngest age group being subjects less than 25 years old. They reported that older patients had a significantly lower chance of tooth survival [16]. Lazarski et al. included 14- to 90-year-old subjects and reported that the incidence of extraction increases with patient age [19]. Caplan and Weintraub found that older patients were more likely to receive extractions of ETT per 10-year increase in age [20]. The patients included were at least 21 years of age at the time of treatment.

Because our study included only a pediatric population, our findings were different from those studies stating that ETT failure increases with age [13, 16, 17, 19, 20]. In our study, younger age at treatment was associated with lower survival. ETT were more likely to survive when NSRCTs were performed at ages 15–18 years than at younger ages. Our study findings differ but likely complement the findings of prior studies [13, 16, 17, 19, 20]. It could be that survival is lower in the youngest age group (6–14 years) then it increases during late adolescence (15–18 years) and early adulthood and then later drops with increasing age.

NSRCT is a complex and lengthy procedure and requires patient compliance. It would be more difficult for younger patients to tolerate such a procedure. This, along with the anatomy of younger permanent teeth with wide canals and reduced dentinal walls along with immature apices, may be the reasons why fewer ETT would survive in the younger age groups. Age can be considered a surrogate variable for other underlying factors that may influence the outcome of NSRCTs, such as patient compliance, maturity of the apex and thickness of the dentinal walls, all of which are reduced in younger patients. Including vital pulp therapy as a treatment option might increase the chances of tooth survival when pulpal treatment is required at a younger age. The AAE position statement on vital pulp therapy has suggested the use of less invasive procedures even in cases diagnosed with irreversible pulpitis and that pulpectomy should not be the only treatment option [21].

Other studies, however, did not find any association between survival of ETT and patient age. Dammaschke et al. noticed that failure increased with age but it was not statistically significant [11]. Their study only included adult patients (18–74 years of age). Mareschi et al. and Swartz et al. have reported that age at treatment does not have a significant effect on treatment success [12, 22]. Mareschi et al. included adult patients only [14], while Swartz examined all age groups, with the youngest group being less than 10 years and the oldest group being 70–79 years [22].

Among our study sample of children, the 3-year survival rate was estimated to be 83.5% for 15- to 18-year-olds, 70.6% for 12- to 14-year-olds and 61.9% for 6- to 11-year-olds. In the total sample, the average survival rate was 76.2%. Using data from a major German national health insurance company, Raedel et al. reported that 84.3% of more than 500,000 ETT survived at 3 years, similar to our 15- to 18-year-old survival rate [23]. Analyzing the data of insured dental patients, Lazarski et al. reported that 94.4% of more than 100,000 ETT survived over an average follow-up period of 3.5 years [19]. In both of these studies, failure was determined when the following interventions were recorded: retreatment, apical surgery, and extraction [19, 23]. In addition to the above, our study also included symptomatic ETT in the failure category. This difference in the survival criteria used along with the different practices and patient types may lead to a lower general survival rate in our study.

In our study, 14.9% of 424 ETT failed throughout the study period with a median survival time of 77 months. Failures occurred most among 6- to 11-year-olds (26.3%) followed by 12- to 14-year-olds (22.1%) and least among 15- to 18-year-olds (10.6%). Cheung reported that 44% of 251 ETT failed, with a median survival time of 113 months [15]. Another study reported that 52% of 608 ETT failed, with a median survival time of 111 months [18]. Their definition of failure was similar to ours, except we did not include the presence of periapical radiolucency. Our failure rate was likely lower due to the difference in criteria used to determine failure. We did not consider radiographic status in our analysis, whereas the other studies included asymptomatic teeth with periapical radiolucency in the failure category. Their longer follow-up time would make them more likely to report on failures.

The 5-year survival probability in the above studies was 60–65% [15, 18]. Our 12–14 year group had a similar 5-year survival rate (64.8%), whereas our 6–11 year group had a much lower 5-year survival rate (46.4%). Our 15–18 year group (80%) had a better 5-year survival rate, which is closer to modern endodontics in adults. The NSRCTs in the abovementioned studies were performed during the 1980s and 1990s in a teaching hospital and normal saline was used for irrigation [15, 18]. This would explain the better survival probability in our older age group where modern endodontics were used.

We identified requiring extraction as the major untoward event, followed by retreatment and then apical surgery. In total, 24 ETT (38.1%) were symptomatic; all were planned for either extraction, retreatment or apical surgery except for 2 ETT, where the intended treatment was not recorded. The majority of untoward events occurred within the first 3 years after NSRCT, which was similar to other studies [18, 24]. The incidence of untoward events was reported in other studies to be 3–10.3% [19, 24, 25]. The data of these studies were taken from a large insurance database. Our higher rate of reported untoward events is likely due to the differences in patient and practice type. In the 15–18 year group, however, the incidence of untoward events (10.6%) was similar to a previous study [24]. The studies mentioned above obtained their data from electronic records of insurance companies by searching for codes of untoward events. However, in our case, we followed-up the dental record and radiograph of each patient to determine if any of the untoward events were planned. Not all ETT received the intended treatment. For instance, of the 43 teeth planned for extraction, only 33 had records of extraction performed. Additionally, of the 14 planned retreatments, only 12 were performed. Of the 4 planned apical surgeries, only 3 were performed.

Some studies used tooth retention to report on the presence of ETT disregarding the presence of any symptoms. These studies reported that 83–97% of ETT were retained [2, 11, 13, 19, 25–29]. In our study, 91.7% of ETT were retained, which is comparable to other studies [19, 24, 27, 28]. Some studies reported that 93–97% of subjects had an asymptomatic ETT [30, 31]. In our study, not all failed ETT were symptomatic. Some teeth were extracted for orthodontic reasons or because they were non-restorable. Others were retreated because of an ill-sealed restoration when a patient returned requesting a permanent restoration. An apical surgery was performed because of nonhealing radiolucency while the tooth was symptom free.

In our study we only observed cases for failure. No data were collected regarding caries, fractures, restorations, apex maturity or periapical status of ETT. As our study is a retrospective chart review, this information was not available for all cases. Another weakness of our study is that in our chart review, if a tooth survived, it was assumed that it continued to survive at subsequent time periods unless otherwise stated in the clinical notes or it was absent on a subsequent radiograph. Only if data suggestive of failure were present, the ETT were removed from the survival category at subsequent follow-up time periods. A strength of our approach was that in the failure category, when a tooth was planned for extraction, retreatment or apical surgery, it was considered a failure. However, not all patients received the suggested treatment.

A major limitation in doing this type of research is that the majority (54%) of subjects did not have any clinical or radiographic records after completion of the NSRCT. This could be because the patients did not require any further treatment at the university; hence, the treatment would be considered successful. However, this could also be because the patients sought further treatment elsewhere. Most likely it was a combination of both. At the endodontic clinic of BUGSDM, we have a variable patient pool. Our patients come from internal referrals from other departments within the school or from external referring dental clinics. In the latter, the patients would complete their post endodontic restorative and other treatment at their referring clinic. We did invite our patients for follow-up; however, not all patients chose to attend. Patients should be motivated and educated on the importance of returning for follow-ups after having NSRCTs. Early detection of diseases allows for a better treatment, prognosis and overall dental experience.

## Conclusions

Age at treatment was a significant factor in determining the risk of ETT failure among children aged 6–18 years. The 5-year survival probability of ETT was 80.0% for 15- to 18-year-olds, 64.8% for 12- to 14-year-olds, and 46.4% for 6- to 11-year-olds. The average 5-year survival of ETT for all age groups was 69.1%. Our results suggest that the longer NSRCT is prevented, through proper oral hygiene measures, preventive dental care and less invasive procedures, the better the likelihood of survival of ETT in children. Vital pulp therapy should be considered over NSRCT when possible in younger children.

## Data Availability

The datasets used and/or analyzed during the current study are available from the corresponding author on reasonable request.

## Abbreviations

NSRCT: Nonsurgical root canal treatment
ETT: Endodontically treated teeth
BUGSDM: Boston University Henry M. Goldman School of Dental Medicine
HR: Hazard ratio
CI: Confidence interval
KM: Kaplan–Meier
